# An open source infrastructure for managing knowledge and finding potential collaborators in a domain-specific subset of PubMed, with an example from human genome epidemiology

**DOI:** 10.1186/1471-2105-8-436

**Published:** 2007-11-09

**Authors:** Wei Yu, Ajay Yesupriya, Anja Wulf, Junfeng Qu, Muin J Khoury, Marta Gwinn

**Affiliations:** 1 National Office of Public Health Genomics, Coordinating Center for Health Promotion, Centers for Disease Control and Prevention, Atlanta, GA, USA; 2 Department of Information Technology, Clayton State University, Atlanta, GA, USA

## Abstract

**Background:**

Identifying relevant research in an ever-growing body of published literature is becoming increasingly difficult. Establishing domain-specific knowledge bases may be a more effective and efficient way to manage and query information within specific biomedical fields. Adopting controlled vocabulary is a critical step toward data integration and interoperability in any information system. We present an open source infrastructure that provides a powerful capacity for managing and mining data within a domain-specific knowledge base. As a practical application of our infrastructure, we presented two applications – Literature Finder and Investigator Browser – as well as a tool set for automating the data curating process for the human genome published literature database. The design of this infrastructure makes the system potentially extensible to other data sources.

**Results:**

Information retrieval and usability tests demonstrated that the system had high rates of recall and precision, 90% and 93% respectively. The system was easy to learn, easy to use, reasonably speedy and effective.

**Conclusion:**

The open source system infrastructure presented in this paper provides a novel approach to managing and querying information and knowledge from domain-specific PubMed data. Using the controlled vocabulary UMLS enhanced data integration and interoperability and the extensibility of the system. In addition, by using MVC-based design and Java as a platform-independent programming language, this system provides a potential infrastructure for any domain-specific knowledge base in the biomedical field.

## Background

Published literature databases are a major information source for generating scientific hypotheses and conducting evidence-based reviews [1]. PubMed/Medline is the largest published literature repositories in the biomedical world, containing more than 15 million citations from about 5000 journals [2]. The ever-increasing quantity of published literature creates challenges for searching and data-mining. Many efforts have attempted to improve the search capacity and performance of PubMed, for example, by creating an alternative, user-friendly Web interface [3], adopting a semantics-based ranking algorithm [4], or using a BLAST-style text similarity search algorithm [5]. Several biomedical research fields have also generated domain-specific, Web-based information resources by collecting and curating PubMed citations and other data relevant to their interests [6-8]. These smaller, more specific information sources can be more easily queried and used for domain-specific data mining.

The human genome epidemiology literature database [9] is a domain-specific, published-literature database created originally in 2001, sponsored by the National Office of Public Health Genomics at the Centers for Disease Control and Prevention. By collecting and curating citations from PubMed that report epidemiologic analyses of gene-disease associations, the database facilitates meta-analyses in the rapidly emerging field of human genome epidemiology (HuGE)[10]. The database currently contains more than 30,000 citations with more than 6,000 new articles added each year [11].

We present an open source infrastructure for managing and querying domain-specific data (Literature Finder) and investigator information (Investigator Browser) available in PubMed abstracts, along with a data management and curating tool set. We illustrate these functions using a knowledge base system called HuGE Navigator [12] which was built upon this open source infrastructure. By integrating the Unified Medical Language System (UMLS) into our open source package, we provide a novel IT infrastructure that facilitates data integration, interoperability, and allows for future expansion to include additional applications.

## Results

### Implementation

The Web-based infrastructure for this system was designed to manage a local collection of PubMed literature and generate investigator profiles based on authorship and affiliation information. The main objectives of the design were data standardization, automatic capacity for data manipulation, modularity and scalability of the system, and a user-friendly Web interface.

#### Data management implementation

The UMLS [13] contains more than 100 vocabularies from biomedical fields; the many synonyms and variants for each unique concept are linked with a UMLS concept unique identifier (CUI). Indexing the literature using UMLS CUIs enhances interoperability and integration of the data, while increasing the sensitivity of data retrieval and allowing for robust free text searching and system extensibility.

To increase the granularity of relationships among the millions of unique concepts collected in UMLS, we used the Medical Subject Headings (MeSH) hierarchy tree to establish parent-child relationships. MeSH [14] indexed by PubMed staff are converted to UMLS CUIs automatically, reducing the manpower needed in the curating process.

We enriched the gene information in the UMLS metathesaurus by incorporating data downloaded from Entrez Gene records [15], substituting Entrez Gene IDs for the UMLS CUIs. Although HUGO, a nomenclature for human genes [16], is one of the controlled vocabularies in the UMLS, we found that Entrez Gene was more comprehensive than HUGO, including more gene aliases and additional genetic information, such as chromosome location and OMIM ID.

To improve the performance of the system, we designed a dynamic data subset-creating process for the external datasets. The 6 million records in the UMLS concept-lookup table could create performance issues if queried directly. Even after removing non-English and retired terms, the table contained 3 million records. Because of the multidisciplinary nature of HuGE research, we could not further subset the UMLS metathesaurus by semantic type. Since not all 3 million UMLS terms will be used in literature indexing, we developed a UMLS concept subset-creating script as part of the curating utilities. This script populates the subset table automatically and dynamically by adding any newly encountered terms into the UMLS subset table in the database when PubMed records are uploaded or updated (see Curating utility implementation section). Applying this process dramatically reduced the size of the UMLS subset table to about 23,000 records, significantly improving performance. The same mechanism was applied to the MeSH hierarchy data, creating subsets that are used to retrieve children terms for the query.

#### Application feature implementation

##### Common features

**Figure 1:**
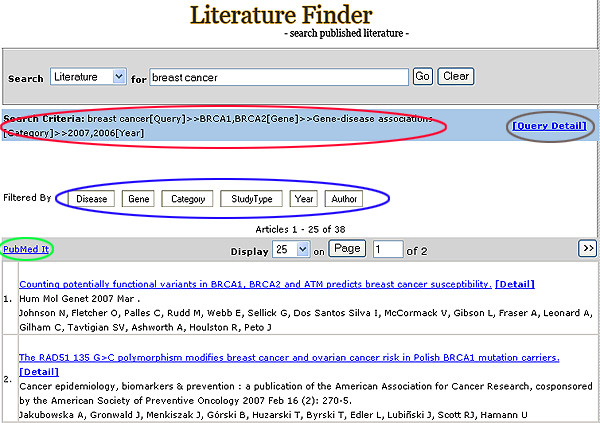
Literature Finder main search page. Brown: Query Detail feature. Red: Traversing tracing feature. Blue: Filtering feature. Green: PubMed It feature.

**Figure 2:**
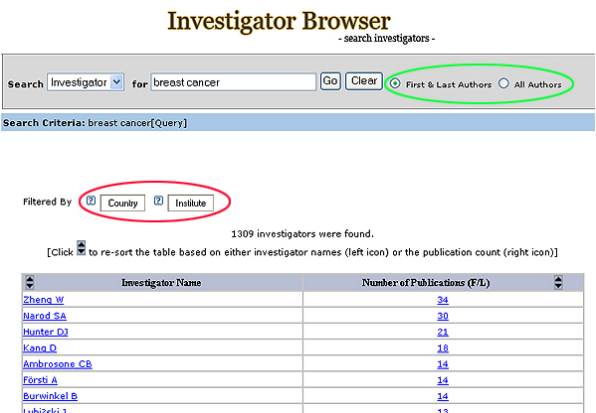
Investigator Browser main search page. Red: Filtering feature. Green: Authorship option.

The user interface was designed to be simple and intuitive, so that the system could be used with minimal instructions. Searches are performed using free text with Boolean operators (or/and) (Figures 1, 2). The Literature Finder and Investigator Browser are cross-referenced anywhere that the publications or authors are displayed.

**Figure 3:**
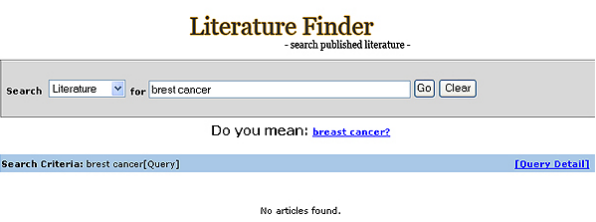
Example of the spelling check feature.

A spelling check feature, equipped with the ESpell [17] NCBI E-Utility that is designed specifically for the biomedical vocabulary, enhances the robustness of free text searching in the system. If a spelling error is found, the system prompts the user with suggested words (Figure 3).

**Figure 4:**
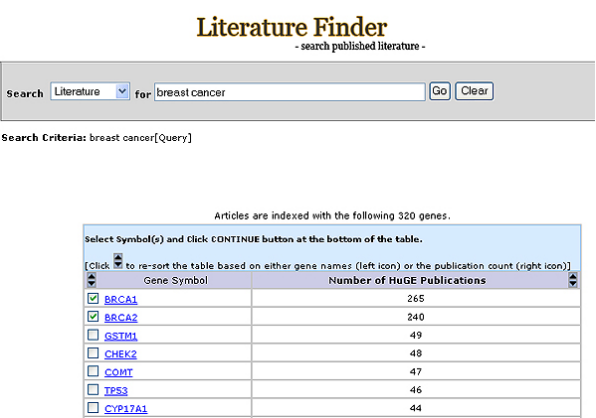
Selection of literature is stratified by gene.

**Figure 5:**
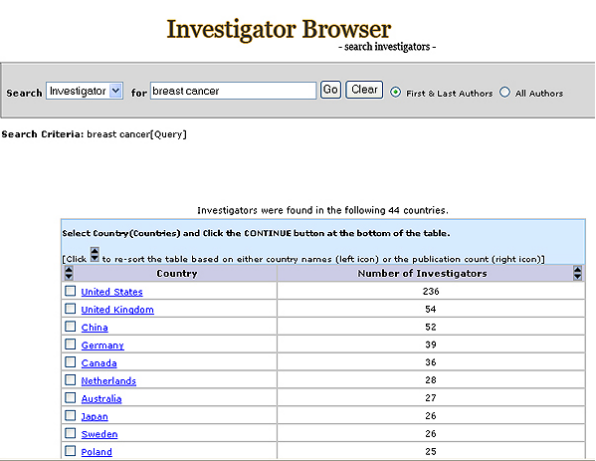
Selection of investigators is stratified by country.

The filtering feature allows further stratification of returned results based on user preference (Figure 4, 5). The filtering process can be performed in an unlimited fashion and the system records all querying and filtering steps (Figure 1).

**Figure 6:**
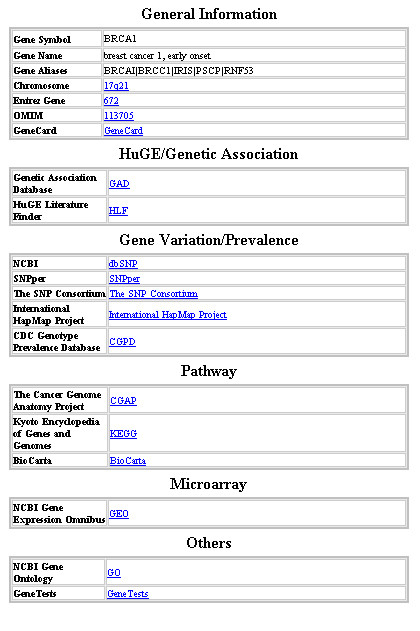
Gene-centered information.

**Figure 7:**
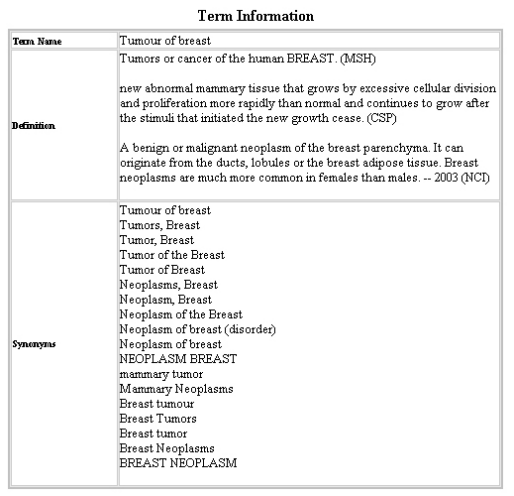
UMLS term description.

Gene-centered information and UMLS concept information can be retrieved from a central page that contains relevant links to general information, genetic association, gene variation/prevalence, and pathway and microarray data (Figure 6). UMLS information includes term definition and synonyms (Figure 7). The user can obtain gene-centered information and UMLS term description information from any page that contains gene or UMLS term links, such as the Query Detail page and intermediate pages for filtering features.

##### Main literature search features

The simple search using the Search Text box returns all available articles that meet the query specification, which can be displayed with 25, 50, or 200 records on a page. The PubMed It feature opens a PubMed page with the returned articles. The user can then search with features provided by PubMed or upload the list of PubMed abstracts into reference software such as Endnote and Reference Manager (Figure 1).

**Figure 8:**
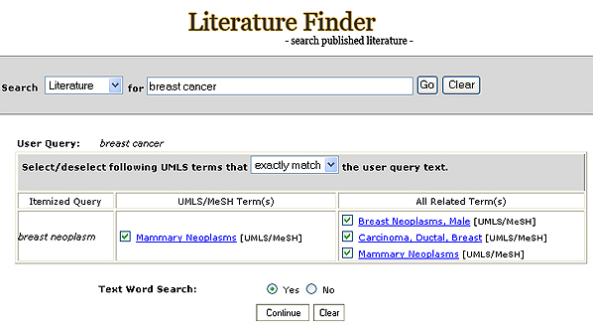
Options for Query Detail feature.

The Query Detail page (Figure 1) provides users the option of modifying the query by selecting or unselecting MeSH terms, including children terms, or turning the text search feature on or off (Figure 8).

Seven classifiers (Disease, Exposure, Gene, Study Type, Category, Year, Author) are available for the filtering feature in the HuGE Literature Finder (Figure 1).

##### Main investigator search features

A list of investigators with their corresponding number of publications is returned based on the user's query in the text search box (Figure 2). The user can choose to display all authors or first and last authors only. The list can be sorted alphabetically by author's name or by the number of publications.

**Figure 9:**
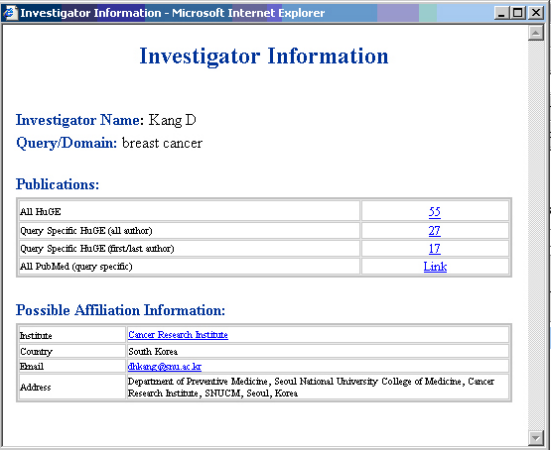
Detail investigator information.

An investigator's profile may be retrieved by clicking on the author's name. If data are available in PubMed, the investigator profile may include the full address, institutional affiliation, country and email address, and the number of publications in the local database as first/last author or any author, and total publications in PubMed (Figure 9).

The filtering feature in the Investigator Browser includes two classifiers: Country and Institute (Figure 2).

#### Curating utility implementation

A number of tools were developed to curate the database automatically. These consist of the following:

The PubMed literature loader automatically uploads records from the PubMed database into the local database using NCBI E-Utilities [18] based on PubMed IDs. Data including the title, abstract, author (first initial, last name), journal (name, volume, issue), publication date (month, year) and affiliation string are used to populate the corresponding database tables.

The MeSH index loader automatically uploads MeSH terms provided in the PubMed record into the database when these terms are available.

The MeSH-UMLS converter converts and maps MeSH terms to the corresponding UMLS CUI.

The UMLS/Entrez Gene subset-generator automatically creates a UMLS/Entrez Gene table with subsets based on terms used in the database.

The MeSH Tree subset-generator automatically creates MeSH Tree table with subsets based on the terms used in the database.

The affiliation parser automatically parses the author affiliation string into full mail address, institution, country and email address, and populates the database with the parsed information. The detailed methodology has been reported [19].

#### Infrastructure implementation

**Figure 10:**
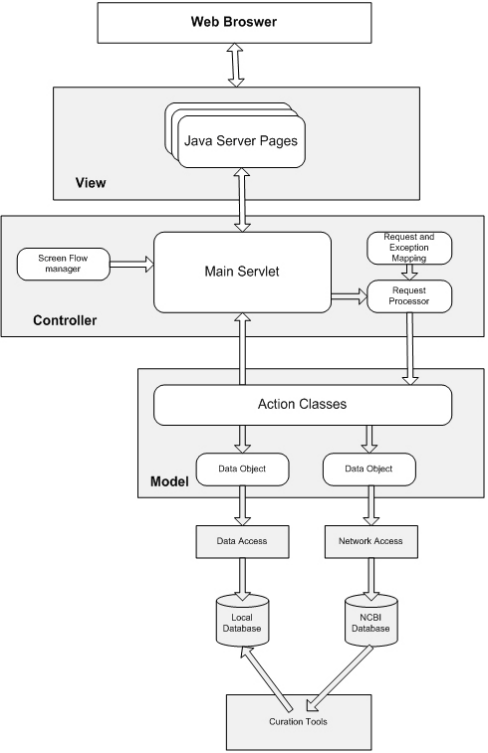
MVC pattern web application architecture overview.

The system was designed using one of the most accepted Web application architectures, the model-view-controller (MVC) pattern [20,21]. This design provides extensive flexibility and scalability because of 1) re-use of model components: the separation of model and view components allows multiple views to use the same enterprise model; 2) easy support for new UI/clients: to support a new UI/client, the view and some controller logic can be simply written and wired into the existing enterprise application; and 3) increased design complexity: the separation of model, view, and controller allows for the introduction of additional classes (Figure 10).

The whole infrastructure can be divided into three discrete modules that are loosely coupled. The data module contains all data in the database; the accessory utility module is responsible for a series of data transactions and manipulations; and the application module includes all the applications in the system. To avoid version control problems, we allow data entities from external data sources (e.g., UMLS Metathesaurus, Entrez Gene, and MeSH Tree) to be updated as needed without an overhaul of the entire system. Each application was built on this model, allowing for seamless navigation and easy plug-in of new applications.

#### Database schema implementation

**Figure 11:**
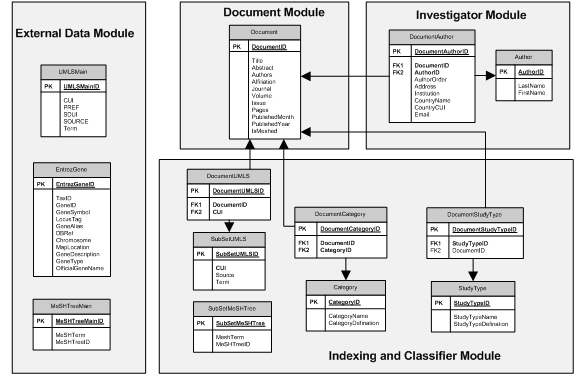
Relational database schema.

A relational database was created based on the requirements of the system. The database design contained the document, investigator, indexing and classifier, and external data modules (Figure 11).

### Information retrieval preliminary evaluation

To test the system's information retrieval performance, we first populated it with 500 randomly selected articles from the human genome epidemiology literature database (HuGE Literature Finder). Independently, two of us (W.Y., A.Y.) assessed all 500 abstracts for relevance to any of the five diseases or the five genes that appear with greatest frequency in the database [9]. All discrepancies were discussed and a final consensus was reached for each article. We then queried the system using the same 10 terms and compared the results with our independent assessment. By using the method described by Zhou, et al. [22], we estimated system recall to be 90% and precision 93%. The formulas to calculate are as followed:

$$Recall=\frac{TP}{TP+FN}$$

$$Precison=\frac{TP}{TP+FP}$$

where TP, FP and FN represent the number of true positives, false positives and false negatives respectively.

### Usability test

We recruited 26 participants to perform a usability test. The participants had diverse backgrounds and included epidemiologists, geneticists, web developers, and graduate students. After a brief introduction and demonstration of the system, each participant used the HuGE Literature Finder to search for the answer to a multiple-choice question. All participants responded to eight statements about usability on a five-point Likert scale. The mode for each statement was calculated to measure central tendency, in keeping with the ordinal nature of Likert scales [23]. Most participants agreed that the system was easy to use, easy to learn, reasonably speedy and effective (Table 1)

**Table 1:** Usability test results.

	**Statements**	**Answers/Total Respondents**					
			
		**SD(1)**	**D(2)**	**N(3)**	**A(4)**	**SA(5)**	**Mode**
1	The interface of this system is pleasant	0/26	0/26	3/26	**16**/26	7/26	**4**
2	I can perform complex searches	0/26	1/26	3/26	**16**/26	6/26	**4**
3	It is easy to find the information I needed	0/26	1/26	0/26	**16**/26	9/26	**4**
4	I feel comfortable using this system	0/26	1/26	2/26	**14**/26	9/26	**4**
5	I can effectively obtain information	0/26	1/26	1/26	**17**/26	7/26	**4**
6	System speed is reasonable	0/26	0/26	1/26	**19**/26	5/26	**4**
7	Easy to learn how to use it	0/26	0/26	0/26	**16**/26	10/26	**4**
8	Overall, I am satisfied with how easy it is to use this system	0/26	0/26	1/26	**13**/26	12/26	**4**

1 = Strongly disagree (SD)

2 = Disagree (D)

3 = Neutral (N)

4 = Agree (A)

5 = Strongly agree (SA)

## Discussion

With advances in Web technology, online searching has become one of the most preferred methods for obtaining information in the health science setting [24]. Although the PubMed/Medline database provides users with a central place to search the biomedical literature, efficient and effective direct searches, using simple key words or complex queries, are often challenging. We have created an open source infrastructure to manage and build a Web-based, domain-specific database from PubMed records. Integrating information from UMLS and Entrez Gene enhances both the sensitivity of the HuGE Literature Finder and the information available to the user. The application infrastructure also provides data mining capacity that automatically extracts investigator profile including mailing address, institution, country and email address from the authorship and affiliation information provided in PubMed abstracts. Recently, developing investigator collaborative networks has become an important agenda in the field of human genome epidemiology, to promote collaborations, facilitate the standardization of study design and analytical methods, confirm findings, and produce systematic reviews [10,25].

There have been many initiatives in the goal of improving the PubMed data retrieval, such as SLIM [3] that enhances the usability of the PubMed, and PubFocus that prioritized the Medline/PubMed record based on the statistical analysis of the query and other factors [4]. A most recent activity is the Semantic Medline developed by the National Library of Medicine that uses the natural language processing technique to predict the semantic relationship of the query to other biologic concepts [26]. As contrast with those initiatives that intend to work on the whole PubMed/Medine, this open source infrastructure is focusing on managing the highly curated PubMed data for a specific biomedical domain and make source codes available to the biomedical community to build their own specialized web-based database easily, simple and intuitive user interfaces increase the usability of information systems. The design of our Web interface accommodates the tendency of most users to search published literature by simple keywords, then filtering down through the retrieved results. The usability test demonstrated that most appreciated this aspect of the interface design.

Modularity and scalability in the MCV-based design of the infrastructure will allow the system to expand easily as needed. Any individual application with specific business logic and requirements can be plugged into the system. We have experimented with this idea by adding supplementary applications, such as other components of HuGE Navigator [12]. A critical feature of this infrastructure application is the use of a robust controlled vocabulary to standardize the data. Because PubMed, Entrez Gene, and UMLS are integrated into the indexing mechanism, the infrastructure of this system is extensible beyond the literature indexing provided by MeSH. For example, integrating laboratory information management into the system would be simple because SNOMED [27], one of the controlled vocabulary collections in UMLS, is suitable for laboratory data. UMLS has successfully demonstrated the ability to map many different controlled vocabularies into the standard vocabulary provided by UMLS concepts [28,29]. The design aims to achieve full integration and interoperability at both the system and data levels.

## Conclusion

The open source system infrastructure presented in this paper provides a novel approach to managing and querying information and knowledge from domain-specific PubMed data. To ensure data integration, interoperability, and system extensibility, we have developed a novel approach to generate dynamically a controlled vocabulary for a specific biomedical domain. A performance evaluation of the system demonstrated high recall and precision. Results of usability testing showed that the Web interface was easy to learn and queries to be completed queries quickly and effectively. The ability to generate a dynamic controlled vocabulary, the MVC-based design, and Java as a platform-independent programming language allow this infrastructure to be used for other domain-specific knowledge bases in the biomedical field.

## Availability and requirements

Project home page: 

The system built upon this open source infrastructure: 

Operating systems: Windows and Linux/Unix

Database: MS SQL server and MySQL

Programming language: Java

Software packages: J2EE 1.4, Hibernate 3.0 and Strut 1.2.9

License: GNU General Public License. This license allows the source code to be redistributed and/or modified under the terms of the GNU General Public License as published by the Free Software Foundation. The source code for the application is available at no charge.

Any restrictions to use by non-academics: None

## Abbreviations

CUI: Concept Unique Identifier

HuGE: Human Genome Epidemiology

HUGO: The Human Genome Organization

MeSH: Medical Subject Heading

MVC: Model-View-Control

OMIM: Online Mendelian Inheritance in Man

UMLS: Unified Medical Language System

## Authors' contributions

WY designed and implemented the infrastructure, wrote the source codes, and drafted the manuscript. AY was involved in the system design and the data analysis and helped in manuscript preparation. AW participated in design of the system evaluation, data collection and analysis. JQ was involved in the system design and configuration, and data management. MG provided advice on the project and revised the draft manuscript. MJK oversaw the project and revised the draft manuscript. All authors read and approved the final document.
